# Thermal Image Sensing Model for Robotic Planning and Search

**DOI:** 10.3390/s16081253

**Published:** 2016-08-08

**Authors:** Lídice E. Castro Jiménez, Edgar A. Martínez-García

**Affiliations:** Laboratorio de Robótica, Institute of Engineering and Technology, Universidad Autónoma de Ciudad Juárez, Cd. Juárez, Chih. 32310, Mexico; 17.lecj@gmail.com

**Keywords:** thermal-imaging, vision, RGB-D, sensing-model, robot-planning, vector-field, fitting-models

## Abstract

This work presents a search planning system for a rolling robot to find a source of infra-red (IR) radiation at an unknown location. Heat emissions are observed by a low-cost home-made IR passive visual sensor. The sensor capability for detection of radiation spectra was experimentally characterized. The sensor data were modeled by an exponential model to estimate the distance as a function of the IR image’s intensity, and, a polynomial model to estimate temperature as a function of IR intensities. Both theoretical models are combined to deduce a subtle nonlinear exact solution via distance-temperature. A planning system obtains feed back from the IR camera (position, intensity, and temperature) to lead the robot to find the heat source. The planner is a system of nonlinear equations recursively solved by a Newton-based approach to estimate the IR-source in global coordinates. The planning system assists an autonomous navigation control in order to reach the goal and avoid collisions. Trigonometric partial differential equations were established to control the robot’s course towards the heat emission. A sine function produces attractive accelerations toward the IR source. A cosine function produces repulsive accelerations against the obstacles observed by an RGB-D sensor. Simulations and real experiments of complex indoor are presented to illustrate the convenience and efficacy of the proposed approach.

## 1. Introduction

In modern robotics, there is an important need to develop robotic technologies to solve real problems in numerous fields. An increasing number of tasks are tied to detecting sources of infra-red (IR) thermal images to accomplish different kinds of autonomous missions. The IR thermal images provide useful intensity data, either in dark or enlighten scenarios which are adequate for robots that carry out a diversity of tasks such as firefighting, autonomous navigation with IR bands, surveillance, search and rescue, underwater missions, volcanic exploration, mining, space exploration, industrial welding, and so forth. An active visual sensor radiates the environment with a form of energy, and the sensor’s receiver detects the energy reflection. Contrastingly, a passive visual sensor solely measures the existing environmental energy (i.e., light intensity). Although commonly passive visual sensors detect visible bands, there is also a number of sensors that detect the IR electromagnetic spectrum, which is an invisible band. These sensors are commonly known as forward-looking IR sensors. These devices are considered thermal IR sensors because they operate in the thermal IR portion of the electromagnetic spectrum, scoping mid-wave ($3\times 10^{3}$ nm–$5\times 10^{3}$ nm) and long-wave ($7\times 10^{3}$ nm–$14\times 10^{3}$ nm) radiant energy sensitive. However, such sensor devices are very costly in the available technological market. In this work, we present the searching/finding problem of an IR thermal radiation source with specific heat range at an unknown location. The robot solely depends on three basic inputs: low-quality and low-resolution IR thermal images, the wheels’ instantaneous rolling speeds, and RGB-D data mapping the obstacles. Therefore, the purpose is to formulate an autonomous searching model for the robot to find a heat source using IR images as feed back.

Among a diversity of applications, there are robotic systems purposed to accomplish tasks where IR thermal sources detection is implicated. The use of IR thermal images in robotic perception is related to carrying out countless missions for detection, inspection, navigation, tracking, localization, and so forth. There exist patrolling autonomous robotic systems purposed to inspect pipelines in thermal power plants for early detection and prevention of leakages [1]. Likewise, robots are instrumented with firefighting monitor systems to extinguish fires in road/railway tunnels [2]. A robotic system for industrial spray deposition that provided self-control to the robot’s course over surfaces with thermal variations was introduced in [3]. In the automotive manufacturing process of radiative paint curing, robot manipulators are based on feed back from UV LEDs detecting heat sources, and an IR thermal camera that measures heat signatures [4]. Fusion algorithms detect fire sources by a micro-car, combining light and temperature sensors on board for tracking gradients of light and heat [5]. Such a work, was designed with an inherent sinusoidal movement to scope $180^{\circ }$ of sight to enhance the limited sensors’ field of view. The work presented experiments in a small area, and proved heuristic confidence levels and conditional probabilistic values as votes to steer the robot.

Unlike previously cited works, we present a robotic planner purposed for exploration missions, which is combined with the IR observations, and directional derivatives for control. Our approach presents results on IR image segmentation, characterization for sensor modeling, numerical analysis with controlled robot’s velocity/acceleration components, and results of the robot’s exploring paths.

Some works reported evaluation of commercial sensors, and IR imaging for data characterization. For instance, in [6] a set of commercial passive sensors was evaluated to determine strengths and weaknesses applied to imaging regions for mud detection. In [7], analysis and preprocessing of thermal–physical features extracted from passive thermal objects were reported. Unlike our approach, we set up a low-cost IR camera, and deduced its measurement models as exact solutions. And, in order to avoid algorithmic complexity, the sensor models provided raw measurement of distance, heat, and pixel intensities.

Several works on tracking and identification using different thermal sensing approaches were reported, for instance, using binocular stereopsis with thermal cameras [8], intense imaging techniques for thermal regions: filtering, segmentation, centroid tracking, morphological operations and edge detection [9], combining an IR thermal camera with a visible band color camera [10], and combining a Kinect’s vision and depth data with a thermopile array sensor [11]. As a difference in our work, we proposed a passive IR visual system that is not combined with another visual technological device, but a simple photographic film. Instead of increasing technological devices, the optical system was characterized to measure 3D positions directly by deducing exponential and polynomial sensing models. Neither, intensive imaging processing, nor matching and optimization techniques, are required. In addition, rather than target-tracking, our system explores and finds thermal targets that are not necessarily present in the field of view. Detection of thermal reflections over objects around is enough to successively find the IR source.

Another major issue developed in this manuscript is the search/explore planner system. For a general survey on robot planning techniques, see [12,13]. Some works have been reported on target-reaching [14], where a micro-car equipped with a photo-transistor is deployed to reach a light in a small arena. Such a work demonstrated results by cumulative probability imaging, and the robot’s path was tracked by using an overhead camera.

The work [15] presented a topological and statistical corridors exploring system using sonar sensors. As a result, the work presented interconnected nodes detected by the ratio of PCA’s eigenvalues, which is an appearance-based method. It was combined with the circle waypoint, which is a path-following method for navigation planning.

In addition, a fuzzy-based target-tracking framework was presented in [16], where a rolling robot used RGB-D depth imaging data for navigation and target-following. It presented empirical results of the RG chromatic space for color identification. And introduced the fuzzy-rules engine to set the kinematic robot’s variables for obstacle avoidance and following a target. Unlike such works, our problem solution does not consider neither, heuristic techniques, nor knowledge in advance of the target to be found. Instead, in order to find the target, a pair of nonlinear equations connects the robot’s positions and the unknown target. Once the system is recursively solved, its solution provides the next local planning. Thereby, our control approach states trigonometric partial differential equations for the robot to avoid the obstacles and reach the IR source.

Furthermore, there are reported works relevant to our specific research in the field of navigation control. For instance, perception-planning using coupled layers for navigation was reported in [17]. Navigation systems comprised of high and low level layers [18], and multi-layered robotic systems [19]. These approaches are designed as architectural software organization, mainly featured by being either global planners, or path generators a priori. Thus, the target position knowledge must be known in advance. Rather, as a main difference, our work approaches a physics-based planner comprised of a system of nonlinear equations solved online and recursively estimating local solutions. Apart of the proposed sensing model, and the planner system, there is a controller that exerts longitudinal and lateral navigation control.

The navigation control strategy provides the robot’s ability to avoid obstacles as it gradually reaches the thermal source. Some traditional approaches are compared in [20]. We can mention the traditional inverse quadratic potential fields combined with state estimation [21], and approaches on time-varying potential fields [22,23]. In addition, other methods are the vector fields techniques using continuous nonlinear functions such as sigmoid [24], exponential [25], trajectories with harmonics [26], polynomial-type to reduce instability and [27], compound adapted trigonometric [28], and steering methods without depth data, but using visual angles [29].

Just for the case of the planner, this part is an incremental contribution of this work. Unlike previous approaches, we span two simple and fast trigonometric functions (sine and cosine) through the gradient operator to geometrically influence two dimensions. We also preserve mathematical simplicity and low computational cost for real-time robotic missions. The proposed navigation function is expressed as a control law that involves the distance obtained by the planner, and the distances measured from the obstacles around, once combined, exert repulsive and attractive behaviors. A similar planner was reported in [30], but solved analytically and combined with exponential vector fields. Such research was applied to find buried pipelines by measuring electrochemical signals on the ground tied to extreme electrical disturbances and steer chattering. Unlike that work, in this paper we provide a faster, computationally simpler solution for the planner through numerical successive approximations spending less than 10 iterations per loop, and numerical precision of $1\times 10^{-6}$.

Finally, deploying the RGB-D for obstacle sensing, the work [31] presented a comprehensive review of recent Kinect-based computer vision algorithms and applications, including preprocessing, tracking and recognition, and 3D mapping. Similar works are reported in [32], where Kinect depth data for indoor mapping applications with high-resolution accuracy was developed. In [33], GPU-based algorithms for real-time RGBD data filtering were implemented. In [34], robot navigation and localization using depth camera was implemented. As a difference in our work, a highly discriminative algorithm for 3D spatial filtering was developed. Local maps are comprised of low density data, which are constrained by a repulsive activation distance. Instead of registering 3D maps, data are computed and organized in the form of 2D LiDAR-like maps, and used to exert vector fields at low computational cost.

The searching problem is tackled by developing three issues: a low-cost home-made thermal IR visual sensor, its IR sensitivity characterization, and formulating its sensing exact solution from sensor measurements. A subtle sensing model calibrates an inexpensive home-made IR visual sensor that infers distance and temperature from an intensity image. The sensing model was formulated with direct and inverse solutions to correlate both the distance and temperature of a heat source. Furthermore, a system of nonlinear equations establishes a search and explore robot’s plan that provides estimations of the source location. The planner equations obtains adjustments based on feed back from the distance-temperature sensor’s observations. The planning equations represent a deterministic system that leads the robot to find the heat radiation source by successive approximations. Nevertheless, we additionally proposed to control the robot by formulating simple but effective trigonometric directional derivatives for navigation control. The planner equations are combined with 2D partial differential equations to produce repulsive and attractive vector fields. Thereby, the robot simultaneously avoid obstacles and find the source of heat emission. This research contributes three major issues listed in ascendant order of importance:

1. An IR visual sensing model that can be applied to any low-cost passive camera. And, it may be adopted as a generalized methodology for different search robotic missions.
2. A local planning model is designed to explore and search goals at unknown locations. The planner is comprised of a time-variant system of nonlinear equations, solved online by a Newton-based method.
3. A navigation controller that is different from other approaches; it is simple, fast, and employs real-time purpose-effective trigonometric functions. The function sine (attractive), and the cosine (repulsive) were formulated as directional derivatives where the angle range [0,⋯,π/2] is transformed into territorial distances.

The paper is organized in the following sections. Section 2 discusses the vision algorithm with adaptive threshold to detect heat emissions regions. Section 3 formulates a sensing model to measure distance from IR intensity images. Section 4 deduces the sensor model closed-form. Section 5 introduces the experimental robot’s scenario. Section 6 establishes a planning system to find the Cartesian location of the heat emissions. Section 7 formulates two trigonometric-based partial differential equations to describe repulsive and attractive behaviors. Section 8 establishes a control navigation function to autonomously reach the IR source. Section 9 discusses the experimental navigational task results. Finally, the conclusions are provided.

## 2. 2D Infra-Red Image Model

**Figure 1:**
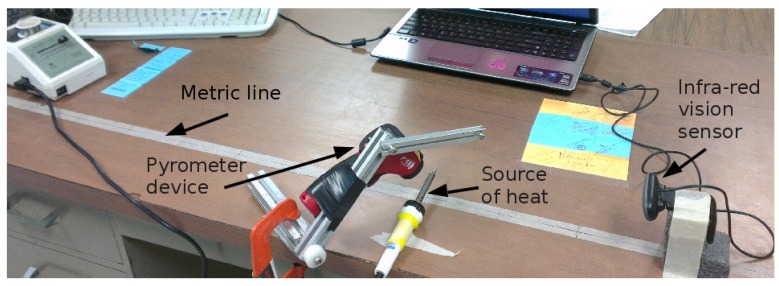
Experimental setup for IR images of sources of heat emission $200{\;}^{\circ }$C–$300{\;}^{\circ }$C.

This section describes the IR visual sensor construction, the image measurement procedure, and the heat regions detection. The home-made sensor device was built with the functionality of an infra-red camera at the lowest possible cost. An ordinary USB camera was modified to capture images in the near/mid infra-red wavelength, with a suitable sized piece of black photographic negative. We researched seven different types of thin sheets of light-sensitive materials to find the best filter for our purpose. We unscrewed the lens assembly from the camera PCB, and removed the small piece of glass used to reflect red light on the back of the lens. Then, we fitted the photographic negative piece between the lens and the CCD. We placed either heat objects or infra-red LEDs and the visual sensor was capable of seeing near infra-red radiation. Other objects of the scene in the normal wavelengths of human vision were not visible because they were all filtered out, except for some red lights at the very red end of the spectrum. The optical features of the photographic film and camera lens are unknown. Thus, different experiments for acquiring images were carried out, ranging from different temperatures between $200{\;}^{\circ }$C and $300{\;}^{\circ }$C. Figure 1 depicts the IR sensor characterization, which consisted of determining a mathematical relationship among the pixel intensities I, the object’s temperatures *T* (${}^{\circ }$C), and its distance *d* (m).

**Figure 2:**
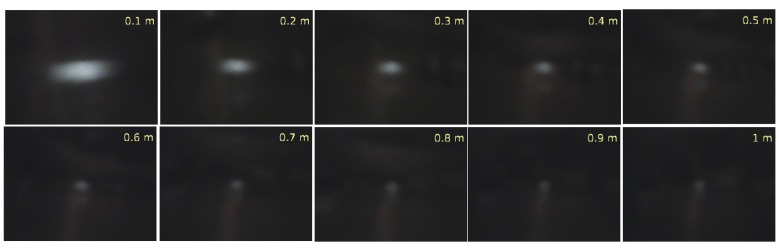
IR intensity images of a thermal source (∼$300{\;}^{\circ }$C) observed at different distances.

A high temperature radiation object (soldering iron) was placed at known distances with millimeter precision, and with known real temperatures externally measured with a highly precise pyrometer device. An illustrative set of IR images in RGB scale is shown in Figure 2, where the heat radiation source (soldering iron) set at ∼$300{\;}^{\circ }$C was placed at successive displacements of 0.1 m. Figure 2 shows IR raw images, where as far as the heat sources move farther, a nonlinear decay of the pixels intensity w.r.t. the source of heat occurred. To search and find a heat source, only a pixel within a region of interest in the image is required. An adaptive threshold value is calculated by statistically ranging the top 5% of pixel intensities (hottest regions).

**Figure 3:**
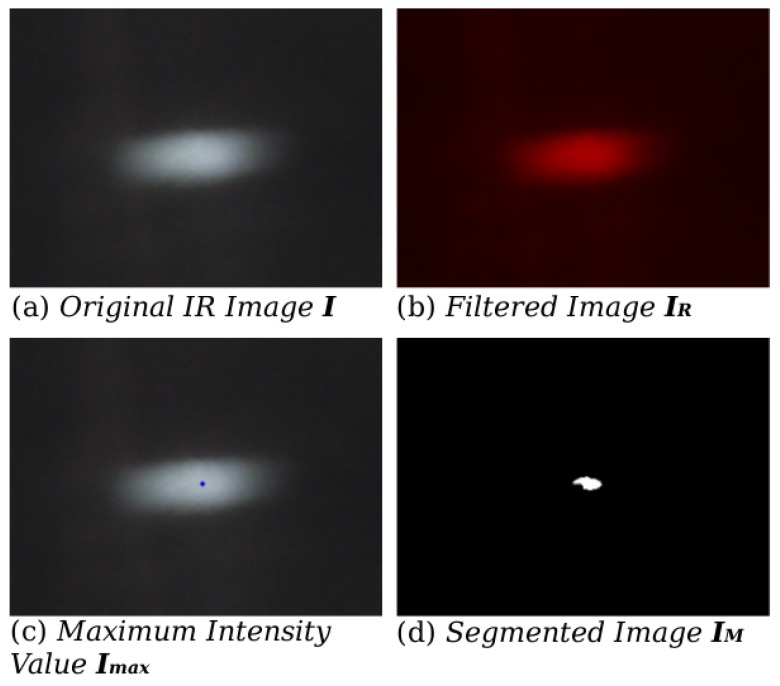
(**a**) Raw IR image (∼$300{\;}^{\circ }$C); (**b**) Red channel image; (**c**) Grey-level hottest point; (**d**) Adaptive threshold results, top 5% intensities region.

Let us define $I_{F}$ as the raw IR image physically filtered by the photographic film, such that $I_{F}\in R^{n\times m\times 3}$, in the 3-color channels RGB as depicted in Figure 3a. Electromagnetic radiation with wave lengths in the range of 5000 nm–5600 nm fall in the near IR spectrum. The raw RGB image $I_{F}$ is filtered out by discriminating the green and blue channels to obtain solely the red channel $I_{R}\in R^{n\times m}$. The near IR wavelength is closer to the red wavelength and it mostly contains the objects’ heat emission of interest Figure 3b. Thus,

(1) $$I_{F}\left(i,j,2\right)=0,\;\mathrm{and}\;I_{F}\left(i,j,3\right)=0,\;\;\forall \;i,j$$

Now, with $I_{R}$, our interest is to detect values of emitted temperatures defined at $300{\;}^{\circ }$C. For the sake of a segmentation process in $I_{R}$, the argument $l_{s}$ that maximizes the intensities in $I_{R}$ is obtained by (see Figure 3c):

(2) $$l_{s}={arg max}_{i,j}I_{R}\left(i,j\right)$$

The argument $l_{s}$ establishes a boundary value of temperatures of interest. Thus, a top factor f=5% rate beneath $l_{s}$ is defined, as well as the least boundary by $l_{i}$:

(3) $$l_{i}=l_{s}\left(1-f\right)$$

Hence, $l_{i}$ is the inferior limit for a segmentation function to detect the pixels representing temperatures of interest. The following definition is expressed:

**Definition 1 (Intensity Region of Interest).**  *The IR image $I_{M}$ contains a segmented region that represents the temperatures of interest*:

(4) $$I_{M}\left(i,j\right)=\left\{\begin{array}{cc} I_{R}\left(i,j\right), & l_{i}\leq I_{R}\left(i,j\right)\leq l_{s}\\ 0, & \text{otherwise} \end{array}\right.$$

Figure 3d illustrates a segmented region obtained by Equation (4) that ranges the top 5% of pixel surrounding $300{\;}^{\circ }$C.

Values obtained by the IR visual sensor were validated by Wein’s law in order to classify the IR sensor sensitive capability and its wave lengths filtering range. The process started from the Planck’s Law describing the intensity radiation per unit area of a source emission. The spectral radiation function L(λ,T) with physical units W/cm${}^{2}\mu$ sr of a black body for a specific wavelength *λ*, and temperature *T* is defined by

$$L\left(\lambda ,T\right)=\frac{C_{1}}{\lambda^{5}e^{\left(\left(C_{2}/\lambda T\right)-1\right)}}$$

where $C_{1}=1.91\times 10^{4}$ (Wμm${}^{4}$/cm${}^{2}$ sr), and $C_{2}=1.428\times 10^{4}$ (μmK). It follows that, the Wien’s law of displacement states that the maximum value of *λ* that causes the peaks curve of the radiation power of a black body, is inversely proportional to its temperature [35].

$$\lambda_{max}=\frac{b}{T}$$

where the constant of displacement of Wien $b=2.8977721\times 10^{-3}$ m·K, and *T* is the temperature (${}^{\circ }$K). Table 1 shows the empirical model of the home-made IR sensor. Data were obtained by the experimental measurements of *T* using a Pyrometer device, the Wien’s Law to calculate *λ*, and the vision algorithm to obtain $I_{M}$ using Equations (1)–(4).

**Figure 4:**
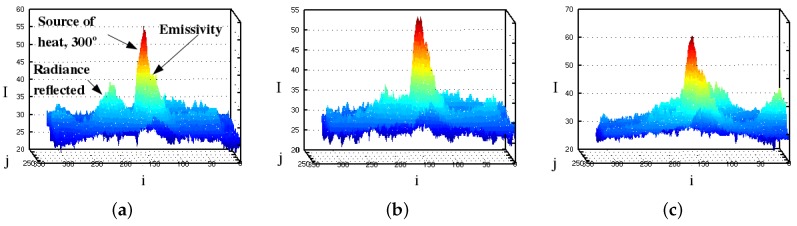
Image mesh of $300{\;}^{\circ }$ IR emissions. (**a**) 0.1 m; (**b**) 0.6 m; and (**c**) 1 m.

In addition, Table 1 empirical values are consistent with the conventional scale of the near IR frequency spectrum and associated temperatures. The IR emissions of Figure 2 (soldering iron) are plotted too as meshes at three different distances by Figure 4. The differences in wave lengths, emissivity, material types, and emission power are all inherently added in the pixel intensity. Some pixels measured both directly from the radiant flux emitted by the source, and surface radiance reflexivity. In Figure 4, columns (*i*) and rows (*j*) are plotted versus pixels intensities I(i,j). The highest peak (red color) contains the intensity pixels bounding to the body’s surface. The proportion of thermal radiation emitted by the object’s surface due to its temperature is known as emissivity. The IR radiance emissivity region is reflected on near surfaces and detected by the sensor.

## 3. Sensor Data and Fitting Models

In this section, the interest is on establishing two sensor models that fit the sensor data. One is a nonlinear polynomial regression model, defined as a function of the image intensity $I_{M}$, in terms of the real distance d(I). Another is an exponential regression model, defined as a function of the image intensity $I_{M}$ in terms of the temperature T(I) [36].

### 3.1. Temperature as a Function of IR Image Intensity

The nonlinear polynomial regression provided a suitable data fit between *T* and $I_{M}$. For practicality, I or $I_{M}$ are indistinctly used hereafter.

**Proposition1.**  *The ideal second degree polynomial model that describes T as a function of $I_{M}$ is:*

(5) $$T\left(I_{i,j}\right)=a_{0}+a_{1}I_{i,j}+a_{2}I_{i,j}^{2}$$

The unknown coefficients $a_{0}$, $a_{1}$ and $a_{2}$ are solved through the least squares estimation, between the ideal model and the sensor data.

(6) $$Sr=\sum_{i=1}^{n}{\left(T_{i}-a_{0}-a_{1}I_{i}-a_{2}I_{i}^{2}\right)}^{2}$$

Thus, the following partial differential equations are stated to be derivable w.r.t. the coefficient of interest:

(7a) $$\frac{\partial Sr}{\partial a_{0}}=-2\sum_{i=1}^{n}\left(T_{i}-a_{0}-a_{1}I_{i}-a_{2}I_{i}^{2}\right)$$

(7b) $$\frac{\partial Sr}{\partial a_{1}}=-2\sum_{i=1}^{n}I_{i}\left(T_{i}-a_{0}-a_{1}I_{i}-a_{2}I_{i}^{2}\right)$$

(7c) $$\frac{\partial Sr}{\partial a_{2}}=-2\sum_{i=1}^{n}I_{i}^{2}\left(T_{i}-a_{0}-a_{1}I_{i}-a_{2}I_{i}^{2}\right)$$

And developing this algebraic process until establishing a model that fits the sensor data trend, the next lemma provides the solutions for the polynomial coefficients.

**Lemma 1 (Polynomial Coefficients of T(I)).**  *The coefficients solution that define the temperature model as a function of the image intensities $T\left(I_{i}\right)=a_{0}+a_{1}I_{i}+a_{2}I_{i}^{2}$, for a total of n observations i is obtained by:*

(8a) $$a_{0}=\frac{\sum T_{i}\left(\sum I_{i}^{2}\sum I_{i}^{4}-{\left(\sum I_{i}^{3}\right)}^{2}\right)+\sum I_{i}T_{i}\left(\sum I_{i}^{2}\sum I_{i}^{3}-\sum I_{i}\sum I_{i}^{4}\right)+\sum I_{i}^{2}T_{i}\left(\sum I_{i}\sum I_{i}^{3}-{\left(\sum I_{i}^{2}\right)}^{2}\right)}{n\sum I_{i}^{2}\sum I_{i}^{4}+2\sum I_{i}\sum I_{i}^{2}\sum I_{i}^{3}-{\left(\sum I_{i}^{2}\right)}^{6}-n{\left(\sum I_{i}^{3}\right)}^{2}-{\left(\sum I_{i}\right)}^{2}\sum I_{i}^{4}}$$

(8b) $$a_{1}=\frac{\sum T_{i}\left(n\sum I_{i}^{4}-{\left(\sum I_{i}^{2}\right)}^{2}\right)+\sum I_{i}^{2}T_{i}\left(\sum I_{i}\sum I_{i}^{2}-n\sum I_{i}^{3}\right)+\sum T_{i}\left(\sum i_{i}^{2}\sum I_{i}^{3}-\sum I_{i}\sum I_{i}^{4}\right)}{n\sum I_{i}^{2}\sum I_{i}^{4}+2\sum I_{i}\sum I_{i}^{2}\sum I_{i}^{3}-{\left(\sum I_{i}^{2}\right)}^{6}-n{\left(\sum I_{i}^{3}\right)}^{2}-{\left(\sum I_{i}\right)}^{2}\sum I_{i}^{4}}$$

(8c) $$a_{2}=\frac{\sum I_{i}^{2}T_{i}\left(n\sum I_{i}^{2}-{\left(\sum I_{i}\right)}^{2}\right)+\sum I_{i}T_{i}\left(\sum I_{i}\sum I_{i}^{2}-n\sum_{i}^{3}\right)+\sum T_{i}\left(\sum I_{i}\sum I_{i}^{3}-{\left(\sum I_{i}^{2}\right)}^{2}\right)}{n\sum I_{i}^{2}\sum I_{i}^{4}+2\sum I_{i}\sum I_{i}^{2}\sum I_{i}^{3}-{\left(\sum I_{i}^{2}\right)}^{6}-n{\left(\sum I_{i}^{3}\right)}^{2}-{\left(\sum I_{i}\right)}^{2}\sum I_{i}^{4}}$$

**Figure 5:**
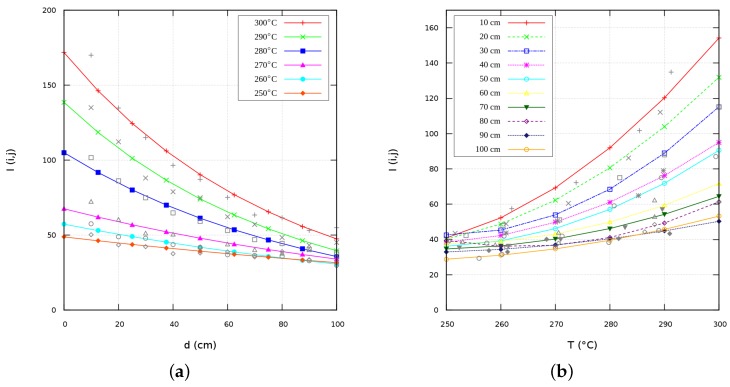
Theoretical-empirical IR sensing models. (**a**) I(d); (**b**) I(T).

The previous polynomial model is validated by the family of temperatures at different distances in Figure 5b. Line-points curves represent the analytical models, while symbol dots are empirical sensor data.

### 3.2. Distance as a Function of IR Image Intensity

A function that calculates the distance *d* between the physical sensor and the IR source is fitted by an exponential regression. Let us assume the general functional form through the next Proposition:

**Proposition** **2.**  *The exponential model that describes d as a function of $I_{i,j}$ is:*

(9) $$d\left(I_{i,j}\right)=\alpha e^{\beta I_{i,j}}$$

Algebraically manipulating the previous expression, it is simplified by applying a logarithm function to both sides of the equality:

(10) ln(d)=ln(α)+βI

Thus, in order to solve for parameters *α* and *β*, a least squares estimation is applied.

(11) $$s_{r}=\sum_{i=1}^{n}{\left(\ln\left(d\right)-\ln\left(\alpha \right)-\beta I\right)}^{2}$$

Next, partially deriving previous equation w.r.t., the unknown parameters:

(12a) $$\frac{\partial s_{r}}{\partial \alpha }=-2\sum_{i=1}^{n}\left[\ln\left(d_{i}\right)-\ln\left(\alpha \right)-\beta I_{i}\right]$$

and

(12b) $$\frac{\partial s_{r}}{\partial \beta }=2\sum_{i=1}^{n}I_{i}\left[\ln\left(d_{i}\right)-\ln\left(\alpha \right)-\beta I_{i}\right]$$

from Equation (12a), the value of ln(α) is obtained and substituted into Equation (12b) in order to produce *β*.

Thus, the following lemma arises,

**Lemma 2.**  *Exponential coefficient of form β:*

(13) $$\beta =\frac{n\sum \ln\left(d_{i}\right)I_{i}-\sum \ln\left(d_{i}\right)\sum I_{i}}{n\sum I_{i}^{2}-{\left(\sum I_{i}\right)}^{2}}$$

Therefore, to validate ranges of temperatures *T*, and distances *d*, Figure 5a depicts the exponential model previously obtained that fits the experimental data. Line curves are deterministic models, while symbol points are empirical data.

## 4. Nonlinear Sensing Model Exact Solution

Mathematical functions fitting the range of the sensor data (Figure 5) are suitable forms to further the analysis in order to deduce new exact solutions. A closed-form function constrained by the variables *T* and *d* is deduced, both terms are deployed to infer a new analytical direct/inverse solution. Initially, let us consider both previous decoupled sensing models:

(14) $$d\left(I_{i,j}\right)=\alpha \cdot e^{\beta I_{i,j}}$$

and

(15) $$T\left(I_{i,j}\right)=a_{0}+a_{1}I_{i,j}+a_{2}I_{i,j}^{2}$$

Since both models have I redundantly, then drop-off I in both equations:

(16) $$I_{i,j}=\frac{\ln(\frac{d(I_{i,j})}{\alpha })}{\beta }$$

and

(17) $$I_{i,j}=\frac{-a_{1}\pm \sqrt[{2}]{a_{1}^{2}-4\;a_{2}\left[a_{0}-T\left(I_{i,j}\right)\right]}}{2a_{2}}$$

and by equating both resulting equations, variables *d* and *T* are expressed mutually as functions of each other:

(18) $$\frac{\ln(\frac{d(I_{i,j})}{\alpha })}{\beta }=\frac{-a_{1}\pm \sqrt[{2}]{a_{1}^{2}-4\;a_{2}\left[a_{0}-T\left(I_{i,j}\right)\right]}}{2a_{2}}$$

and raised to the second power:

(19) $${\left(\frac{\ln\left(\frac{d(I_{i,j})}{\alpha }\right)\cdot 2a_{2}}{\beta }+a_{1}\right)}^{2}={\left(\pm \sqrt[{2}]{a_{1}^{2}-4\;a_{2}\left[a_{0}-T\left(I_{i,j}\right)\right]}\right)}^{2}$$

we substitute constant terms by *A* in order to simplify the algebraic process (where *β* prevails constant for an arbitrary temperature):

$$A=\frac{2a_{2}}{\beta }$$

and it follows that:

(20) $${\left(A\;\ln\left(\frac{d(I_{i,j})}{\alpha }\right)+a_{1}\right)}^{2}=a_{1}^{2}-4\;a_{2}\left[a_{0}-T\left(I_{i,j}\right)\right]$$

thus, T(d) is obtained and defined by:

(21) $$T\left(d\left(I_{i,j}\right)\right)=\frac{{\left(A\;\ln\left(\frac{d(I_{i,j})}{\alpha }\right)+a_{1}\right)}^{2}-a_{1}^{2}}{4\;a_{2}}+a_{0}$$

Finally, by replacing *A*, the sensing model is provided in the next proposition,

**Proposition 3 (Temperature as a Function of Distance).**  *The temperature T is obtained by the family of distances d as functions of intensity values I.*

(22) $$T\left(d\left(I_{i,j}\right)\right)=\frac{{\left(\frac{\ln\left(\frac{d(I_{i,j})}{\alpha }\right)\cdot 2a_{2}}{\beta }+a_{1}\right)}^{2}-a_{1}^{2}}{4\;a_{2}}+a_{0}$$

Conversely, to find its inverse solution d(T), we obtain *d* that depends upon *T*, starting from Equation (18). Thus, multiplying both sides of equation by *β*’:

(23) $$\ln\left(\frac{d(I_{i,j})}{\alpha }\right)=\frac{-a_{1}\pm \sqrt[{2}]{a_{1}^{2}-4\;a_{2}\left[a_{0}-T\left(I_{i,j}\right)\right]}}{2a_{2}}\cdot \beta$$

and applying the exponential product to both sides of the previous equation:

(24) $$\frac{d(I_{i,j})}{\alpha }=e^{\frac{-a_{1}\pm \sqrt[{2}]{a_{1}^{2}-4\;a_{2}\left[a_{0}-T\left(I_{i,j}\right)\right]}}{2a_{2}}\cdot \beta }$$

The distance *d* is defined by the next proposition,

**Proposition 4 (Distance as a Function of Temperature).**  *The distance d is obtained by the family of temperatures T in terms of intensity values I.*

(25) $$d\left(T\left(I_{i,j}\right)\right)=\alpha e^{\frac{-a_{1}\pm \sqrt[{2}]{a_{1}^{2}-4\;a_{2}\left[a_{0}-T\left(I_{i,j}\right)\right]}}{2a_{2}}\cdot \beta }$$

In the form provided by the authors, these sensing models work for a specific curve. Notice that a fitted curve model behaves according to the sensor being characterized, and such a model implicitly includes all effects of temperature values and distance, material emissivity and so forth. For the specific case presented in this work, if the photographic film is replaced by another material, then the polynomial and exponential coefficients obtained from mathematical regressions have to be re-calculated.

Furthermore, the real measured distance d(I) of the thermal source w.r.t. the robot’s sensor is enough information to infer the thermal source in 3D space coordinates ${\left(d_{x},d_{y},d_{z}\right)}^{⊤}$. The model is obtained through spherical coordinates by:

(26) $$\left(\begin{array}{c} d_{x}\\ d_{y}\\ d_{z} \end{array}\right)=d\left(I\right)\left(\begin{array}{c} \sin\left(\Delta \phi_{0}\right)\cos\left(\Delta \phi_{1}\right)\\ \sin(\Delta \phi_{1})\\ \cos\left(\Delta \phi_{0}\right)\cos\left(\Delta \phi_{1}\right) \end{array}\right)$$

where the azimuth angle $\Delta \phi_{0}$, and the elevation angle $\Delta \phi_{1}$ w.r.t. the center of the IR-camera location are defined by:

(27) $$\Delta \phi_{0}=\frac{\phi_{0}}{C}\eta_{C};\;\Delta \phi_{1}=\frac{\phi_{1}}{R}\eta_{R}$$

where *C* and *R* are the image plane number of columns and rows respectively. Likewise, $\eta_{C}$, and $\eta_{R}$ are the pixel coordinates of the centroid that maximizes the heat region.

## 5. Experimental Scenario

**Figure 6:**
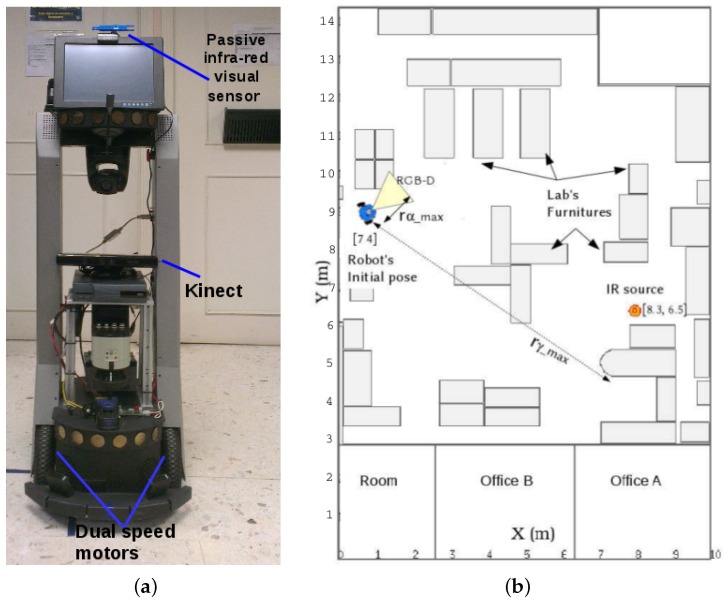
(**a**) Experimental self-contained robotic platform; (**b**) Layout of the Robotics Lab, and experimental configuration with an IR thermal source of 850 nm at (7 m, 4 m).

This section describes the robotic platform, the experimental scenario, and the type of search and explore missions that sustained this work through diverse indoor experiments. This work deployed a Peoplebot platform (Mobile Robotics${}^{TM}$), which is depicted in Figure 6a. This type of robotic platform is commercially instrumented with numerous sensing devices, but none of them were deployed for this work. The home-made IR sensor and the RGB-D device were added to accomplish this work. In addition, the dual in-wheel encoders were deployed to measure the robot’s position $x_{r}={\left(x_{r},y_{r}\right)}^{⊤}$.

The experiments were carried out in the Robotics Laboratory (Juárez city, México), which is a complex dynamic environment with multiple pieces of furniture, equipment, and people around. The Lab’s working area measures 10m×11m, and its layout is depicted in Figure 6b. The layout is an accurate draw, and metrically resembles the Lab’s objects organization. Although this layout illustrates only one robot’s starting position, a diverse grouping of robot’s initial locations was used to yield multiple pathways.

The global coordinate system was established at the layout down left-sided. As for the parameter $r_{\alpha_{max}}$, it is the maximal territorial distance used by the robot to avoid collisions. Likewise, the parameter $r_{\gamma_{max}}$ is the territorial distance for attraction towards the IR emission source.

**Figure 7:**
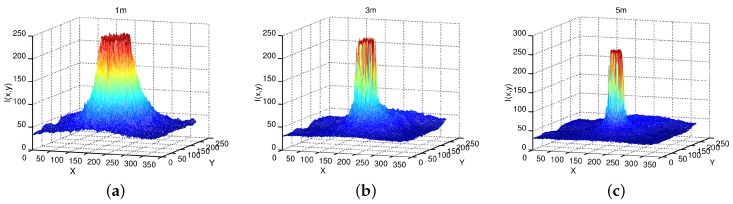
Sensor observation of a source of near-IR radiation of 850 nm, and a radiance power of 75.5 mW, at (**a**) 1 m; (**b**) 3 m and (**c**) 5 m.

We implemented a safety high radiance device that emitted near-IR emissions beyond 10 m using IR-LEDs. The device emits nearly 850 nm (IR electromagnetic spectrum) that surrounds $700{\;}^{\circ }$C, with a radiance power of 75.5 mW per steradian. Figure 7 shows some near-IR image surfaces measured at different distances using the same home-made IR camera. The near-IR source device was directly oriented towards the robot’s sensor for these measurements. The image’s coordinates *X* are the columns, *Y* are the rows, and data are plotted w.r.t. pixels intensity I(x,y).

## 6. Planning Equations for IR-Source Searching

**Figure 8:**
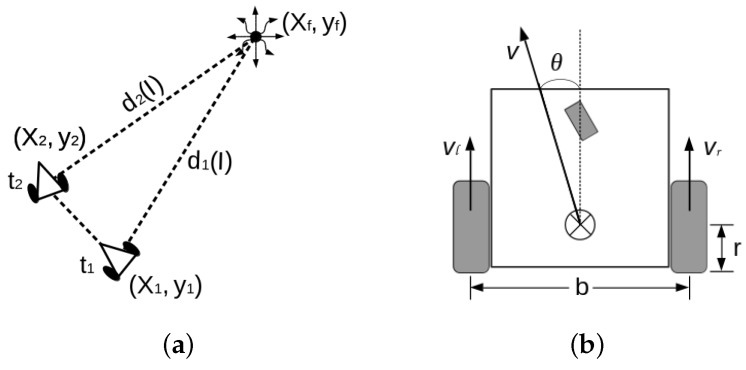
(**a**) Search planning strategy using pairs of successive IR-image observations; (**b**) Robot’s kinematics parameters.

Let us assume that two successive positions $\left(x_{1},y_{1}\right)$ and $\left(x_{2},y_{2}\right)$ are known. And a final value $\left(x_{f},y_{f}\right)$) is to be estimated, see please planning and robot kinematic parameters of Figure 8. The robot passes through $\left(x_{1},y_{1}\right)$ and $\left(x_{2},y_{2}\right)$ in a finite period of time $\Delta t=t_{2}-t_{1}$, measuring $d_{1}\left(T_{1}\right)$ and $d_{2}\left(T_{2}\right)$ (Equation (25)) at each position respectively.

The search/find planning scheme is established by the next general equation, which is established according to Figure 8a. All coordinates deployed in the planning framework are managed in global coordinates. Nevertheless, the observation distances $d{\left(T\right)}_{1,2}$ are sensed in the robot’s fixed frame.

(28) $${\left(x_{t}-x_{f}\right)}^{2}+{\left(y_{t}-y_{f}\right)}^{2}=d_{t}^{2}\left(T\left(I\right)\right)$$

Therefore, Equation (28) is spanned by the next axiom into a system of two independent equations that describe the exploring motion.

**Axiom 1 (System of Nonlinear Equations).**  *Redefining $d_{1,2}^{2}=d_{1,2}^{2}\left(T\left(I\right)\right)$; in time $t_{1}$ the expression $f_{1}\left(x,y\right)$ is established by:*

(29a) $$f_{1}\left(x,y\right)={\left(x_{1}-x_{f}\right)}^{2}+{\left(y_{1}-y_{f}\right)}^{2}-d_{1}{\left(T\left(I_{1}\right)\right)}^{2}=0$$

*and in time $t_{2}$ the expression $f_{2}\left(x,y\right)$ is stated by:*

(29b) $$f_{2}\left(x,y\right)={\left(x_{2}-x_{f}\right)}^{2}+{\left(y_{2}-y_{f}\right)}^{2}-d_{2}{\left(T\left(I_{2}\right)\right)}^{2}=0$$

If more robot’s positions are used, the number of equations or constraints increase. Hence, the system will become overdetermined and more equations will constraint the two unknowns (degrees of freedom) of the system. Although, for this specific case it is possible to deduce an analytical solution, we proposed a numerical recursive successive approximation method. Both, analytical and numerical calculations have been proved, and we conclude that the numerical modality is faster and more feasible for online implementations. Therefore, $x_{f}$ and $y_{f}$ are solved by the multivariate version of the Newton-Raphson method stated by the next equation:

(30) $$f_{i}\left(x+\delta x\right)=f_{i}\left(x\right)+\sum_{j=1}^{N}\frac{\partial f_{i}}{\partial x_{j}}\delta x_{j}$$

separating the vector components into two equations:

(31) $$f_{1}{\left(x,y\right)}_{t+1}=f_{1}\left(x,y\right)+\left(x_{t+1}-x_{t}\right)\frac{\partial f_{1}\left(x,y\right)}{\partial x}+\left(y_{t+1}-y_{t}\right)\frac{\partial f_{1}\left(x,y\right)}{\partial y}$$

and

(32) $$f_{2}{\left(x,y\right)}_{t+1}=f_{2}\left(x,y\right)+\left(x_{t+1}-x_{t}\right)\frac{\partial f_{2}\left(x,y\right)}{\partial x}+\left(y_{t+1}-y_{t}\right)\frac{\partial f_{2}\left(x,y\right)}{\partial y}$$

Therefore, our purpose is to iteratively reach a zero value for each prediction function $f_{1}{\left(x,y\right)}_{t+1}$ and $f_{2}{\left(x,y\right)}_{t+1}$, as they are the real roots. Thus,

(33) $$f_{1}\left(x,y\right)+\left(x_{t+1}-x_{t}\right)\frac{\partial f_{1}\left(x,y\right)}{\partial x}+\left(y_{t+1}-y_{t}\right)\frac{\partial f_{1}\left(x,y\right)}{\partial y}=0$$

and

(34) $$f_{2}\left(x,y\right)+\left(x_{t+1}-x_{t}\right)\frac{\partial f_{2}\left(x,y\right)}{\partial x}+\left(y_{t+1}-y_{t}\right)\frac{\partial f_{2}\left(x,y\right)}{\partial y}=0$$

By reorganizing algebraically:

(35) $$x_{t+1}\frac{\partial f_{1}\left(x,y\right)}{\partial x}+y_{t+1}\frac{\partial f_{1}\left(x,y\right)}{\partial y}=-f_{1}\left(x,y\right)+x_{t}\frac{\partial f_{1}\left(x,y\right)}{\partial x}+y_{t}\frac{\partial f_{1}\left(x,y\right)}{\partial y}$$

In its matrix form, $x={\left(x,y\right)}^{⊤}$, and $f={\left(f_{1},f_{2}\right)}^{⊤}$,

(36) $$f_{i}\left(x_{t}\right)+J\left(x_{t+1}-x_{t}\right)=0$$

the next Cartesian components calculation is provided by:

(37) $$x_{t+1}=x_{t}-J^{-1}\cdot f_{i}\left(x_{t}\right)$$

where the Jacobian matrix is defined by:

(38) $$J=2\left(\begin{array}{cc} \left(x_{1}-x_{f}\right) & \left(y_{1}-y_{f}\right)\\ \left(x_{2}-x_{f}\right) & \left(y_{2}-y_{f}\right) \end{array}\right)$$

likewise, its inverse solution is stated by:

(39) $$J^{-1}=\frac{2}{\det(J)}\cdot \left(\begin{array}{cc} \left(y_{2}-y_{f}\right) & -(y_{1}-y_{f})\\ -(x_{2}-x_{f}) & \left(x_{1}-x_{f}\right) \end{array}\right)$$

By substituting the Jacobian to predict the next Cartesian components:

(40) $$\left(\begin{array}{c} x_{t+1}\\ y_{t+1} \end{array}\right)=\left(\begin{array}{c} x_{t}\\ y_{t} \end{array}\right)-\frac{2}{\det(J)}\cdot \left(\begin{array}{cc} \left(y_{2}-y_{f}\right) & -(y_{1}-y_{f})\\ -(x_{2}-x_{f}) & \left(x_{1}-x_{f}\right) \end{array}\right)\cdot \left(\begin{array}{c} f_{1}\left(x_{t},y_{t}\right)\\ f_{2}\left(x_{t},y_{t}\right) \end{array}\right)$$

The determinant of the Jacobian matrix is obtained in the following manner:

(41) $$\det\left(J\right)=\frac{\partial f_{1}}{\partial x}\cdot \frac{\partial f_{2}}{\partial y}-\frac{\partial f_{2}}{\partial x}\cdot \frac{\partial f_{1}}{\partial y}$$

algebraically developing, simplifying, and reorganizing:

(42) $$\det\left(J\right)=4[x_{1}y_{2}-x_{2}y_{1}+x_{f}\left(y_{1}-y_{2}\right)+y_{f}\left(x_{2}-x_{1}\right)]$$

**Proposition** **5. (Nonlinear Planning Solution)**  *The solution for $\left(x_{i+1},y_{i+1}\right)$ is recursively estimated by:*

(43) $$x_{t+1}=x_{t}-\frac{1}{2}\left(\frac{f_{1}\left(x_{t},y_{t}\right)\left(y_{2}-y_{f}\right)-f_{2}\left(x_{t},y_{t}\right)\left(y_{1}-y_{f}\right)}{x_{1}y_{2}+x_{2}y_{1}+x_{f}\left(y_{1}-y_{2}\right)+y_{f}\left(x_{2}-x_{1}\right)}\right)$$

*and*

(44) $$y_{t+1}=y_{t}-\frac{1}{2}\left(\frac{f_{2}\left(x_{t},y_{t}\right)\left(x_{1}-x_{f}\right)-f_{1}\left(x_{t},y_{t}\right)\left(x_{2}-x_{f}\right)}{x_{1}y_{2}+x_{2}y_{1}+x_{f}\left(y_{1}-y_{2}\right)+y_{f}\left(x_{2}-x_{1}\right)}\right)$$

In its recursive process, the coordinates $\left(x_{t},y_{t}\right)$ are expected to gradually reach the value $\left(x_{f},y_{f}\right)$. Being the best numerical approximated estimation of the thermal source position. Therefore, the following remark is stated:

**Remark 1 (Convergence Criterion).**  *The condition criterion for sufficient convergence is defined by the next inequalities expression:*

$$2(\;|x_{1}-x_{f}\left|+\right|x_{2}-x_{f}|\;)<1\;\cap \;2(\;|y_{1}-y_{f}\left|+\right|y_{2}-y_{f}\left|\;\right)<1$$

**Figure 9:**
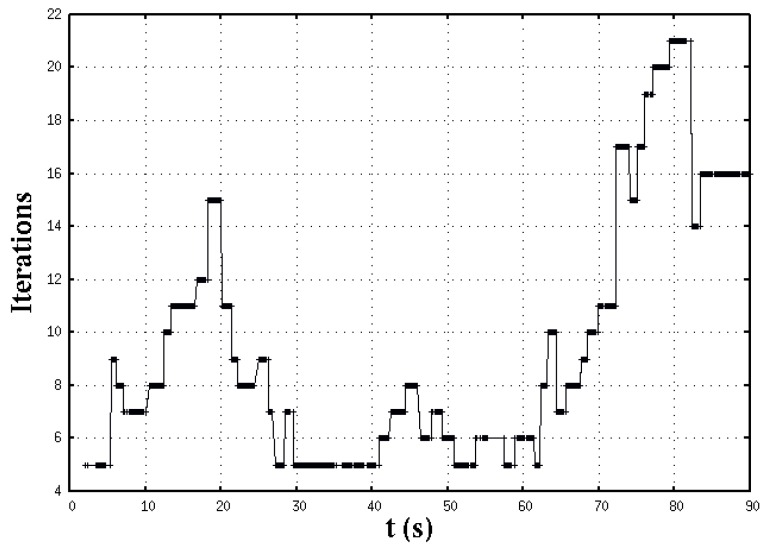
Required number of Newton-Raphson iterations to solve the system of nonlinear equations in each sense-control loop.

During experimental navigation, the nonlinear planning system is solved online with a number of iterations per control loop as the behavior depicted in Figure 9.

## 7. Control by Directional Derivatives

**Figure 10:**
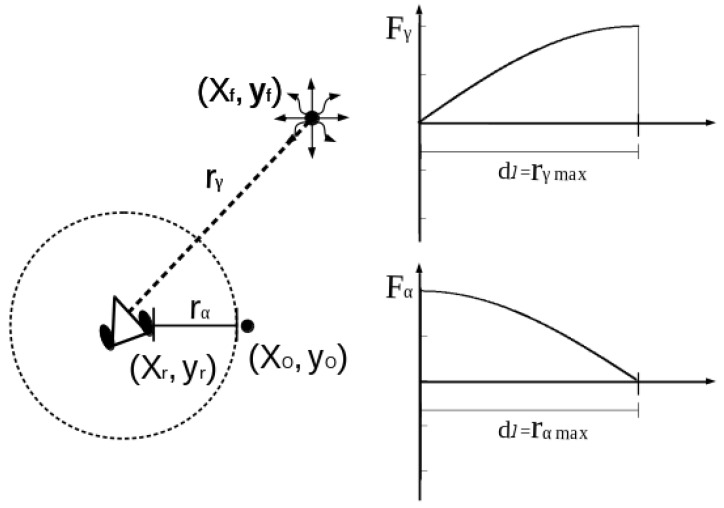
Directional derivatives kinematic parameters and variables.

The planning system deduced in the previous section cannot control the robot’s trajectory, but solely estimates Cartesian observations. In order to deal with real velocities and obstacles detection, this section presents an approach on vector fields to control the robot’s navigation. We proposed the use of the sine and cosine trigonometric functions because of their computational simplicity and their monotonically continuous behaviors in the range $\left[0,..,\frac{\pi }{2}\right]$. The sine function resembles the traditional attractive potential field functions, while the cosine function emulates a kind of repulsive potential field. As a difference from other approaches, we reformulated the sine/cosine function by partial derivation to span them multidimensional. In addition, the territorial/activation distances (attraction or repulsion) have a linear transformation into representative angles. The attractive acceleration poses a function $f_{\gamma }$, an observation distance $d_{l}$ and an activation distance $r_{\gamma_{max}}$. Likewise, the repulsive field deploys an acceleration function $f_{\alpha }$ and an activation distance $r_{\gamma_{\alpha }}$ (see Figure 10).

### 7.1. Attractive Directional Fields

The attractive acceleration $f_{\gamma }$ is defined in terms the actual position (x,y) w.r.t. the IR emission *γ* with coordinates $\left(x_{f},y_{f}\right)$. The next proposition uses the gradient operator applied w.r.t. (x,y), and behaves as illustrated in Figure 10.

**Proposition 6 (Attractive Function).**  *An attractive acceleration $f_{\gamma }$ is a function between the robot position (x,y) and the heat emission γ at $\left(x_{f},y_{f}\right)$.*

(45) $$f_{\gamma }\left(x,y\right)=-\nabla_{x,y}\kappa_{\gamma }r_{\gamma }\cdot \sin\left(\phi_{\gamma }\left(\delta \right)\right)$$

*where $r_{\gamma }$ is the distance to γ that is estimated by the planner calculation. And $\kappa_{\gamma }$ is an acceleration constant gain, set as $\kappa_{\gamma }=1$ for purpose of analysis.*

(46) $$r_{\gamma }=\sqrt[{2}]{x^{2}+y^{2}}=\sqrt[{2}]{{\left(x_{r}-x_{f}\right)}^{2}+{\left(y_{r}-y_{f}\right)}^{2}}$$

The angle $\phi_{\gamma }$ has a direct trigonometric relationship with the sensed distance $\delta_{t}$, which is observed by the IR sensor. If the distance $d_{l}\leq \delta_{t}$, the function (45) is autonomously activated producing an attractive vectors field behavior.

**Definition 2 (Attractive Territorial Distance).**  *The attractive behavior is activated, if the distance $d_{l}\leq \delta_{t}$, such that*:

(47) $$\phi_{\gamma }=\frac{\delta_{t}\pi }{2d_{l}}$$

Therefore, by substituting the functional form of the actual magnitude:

(48) $$f_{\gamma }\left(x,y\right)=-\nabla_{x,y}\;\sqrt[{2}]{{\left(x_{r}-x_{f}\right)}^{2}+{\left(y_{r}-y_{f}\right)}^{2}}\cdot \sin\left(\phi_{\gamma }\left(\delta \right)\right)$$

and by applying the gradient operator $\nabla_{x,y}$, and algebraically simplifying:

(49) $$\frac{\partial f_{\gamma }}{\partial x}=\frac{-\left(x_{r}-x_{f}\right)\cdot \sin\left(\phi_{\gamma }\left(\delta \right)\right)}{\sqrt[{2}]{{\left(x_{r}-x_{f}\right)}^{2}+{\left(y_{r}-y_{f}\right)}^{2}}}$$

and

(50) $$\frac{\partial f_{\gamma }}{\partial y}=\frac{-\left(y_{r}-y_{f}\right)\cdot \sin\left(\phi_{\gamma }\left(\delta \right)\right)}{\sqrt[{2}]{{\left(x_{r}-x_{f}\right)}^{2}+{\left(y_{r}-y_{f}\right)}^{2}}}$$

and replacing $\sqrt[{2}]{{\left(x_{r}-x_{f}\right)}^{2}+{\left(y_{r}-y_{f}\right)}^{2}}$ in its equivalent $r_{\gamma }$ for simplicity:

(51) $$\frac{\partial f_{\gamma }}{\partial x}=\frac{-\left(x_{r}-x_{f}\right)\cdot \sin\left(\phi_{\gamma }\left(\delta \right)\right)}{r_{\gamma }}$$

as well as,

(52) $$\frac{\partial f_{\gamma }}{\partial y}=\frac{-\left(y_{r}-y_{f}\right)\cdot \sin\left(\phi_{\gamma }\left(\delta \right)\right)}{r_{\gamma }}$$

The resulting attractive vector field is stated by the next lemma,

**Lemma 3 (Attractive Directional Vector Field).**  *The acceleration vector towards γ in terms of Cartesian components XY is:*

(53) $$f_{\gamma }\left(x,y\right)=\kappa_{\gamma }\frac{-\sin(\phi_{\gamma })}{r_{\gamma }}\cdot \left(\begin{array}{c} x_{r}-x_{f}\\ y_{r}-y_{f} \end{array}\right)$$

where $\kappa_{\gamma }=1$ is a constant gain in ms${}^{-2}$ for simplicity of analysis.

**Figure 11:**
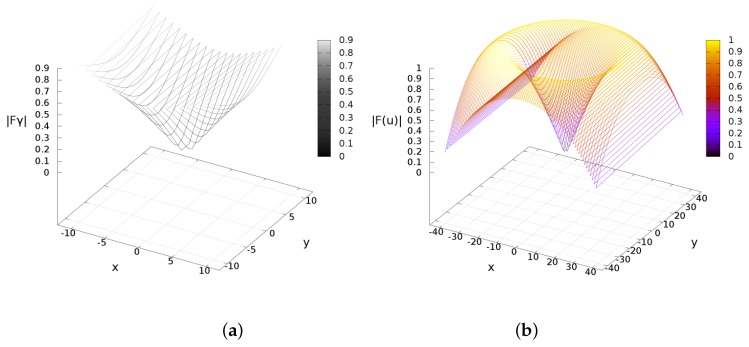
Attractive acceleration behavior plots. (**a**) Effects from $d_{l}\leq r_{\gamma_{max}}$; (**b**) Effects when $d_{l}>r_{\gamma_{max}}$.

**Figure 12:**
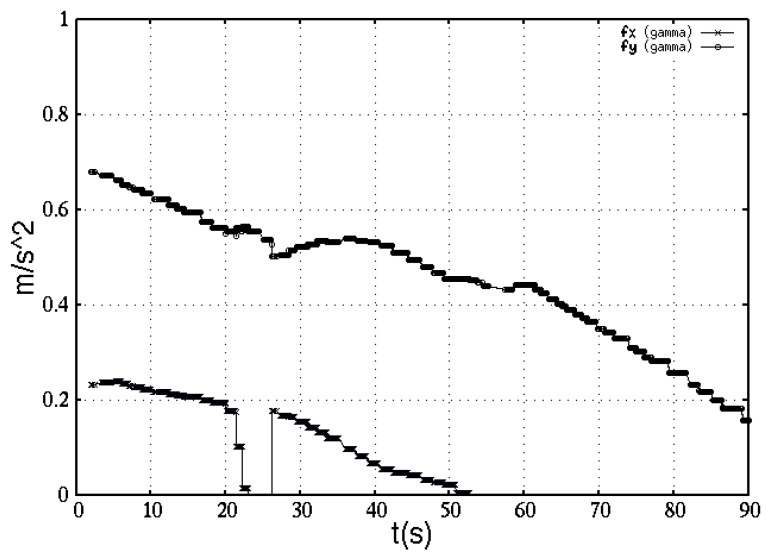
Acceleration components toward a near-IR radiation source.

As a result, Figure 11 depicts a simulation of previous lemma. An experimental pathway developed by the robot to find the near-IR source is represented in terms of the attractive accelerations, Figure 12. The figure shows $f_{\gamma }$ w.r.t. time *t*(s) (experiment duration). Both acceleration components are decreasing as the robot approaches the target location.

### 7.2. Repulsive Directional Fields

In order to avoid collisions with obstacles, an RGB-D sensor (Kinect) is deployed. It is a high-resolution depth and visual (RGB) sensing device available for widespread use. Its complementary capabilities of the depth and visual data facilitate solving fundamental problems in visual perception. Detecting near obstacles that might impede free route navigation is tackled by implementing repulsive directional derivatives. Although, the RGB-D sensor provides 3D depth measurements, the Cartesian sensing model is provided to develop the partial derivative forms as repulsive fields. The RGB-D model establishes the relation of a pixel (i,j) of the Kinect raw image $I_{k}\in R^{w\times h}$ with the robot’s inertial system coordinates (x,y,z). Let the x-Cartesian component be defined by:

(54) $$x=\left(i-w/2\right)\cdot fx^{-1}\cdot z$$

and the y-Cartesian component:

(55) $$y=\left(j-h/2\right)\cdot fy^{-1}\cdot z$$

where fx and fy are the focal lengths of $I_{k}$ expressed in pixels. *z* refers to the sensed depth from the RGB-D sensor. Our interest is to gather and represent a local model of the environment from the 3D points cloud $p_{i}={\left(x,y,z\right)}^{⊤}\in R^{3}$, in particular to determine the nearest obstacles, as provided by the logical premise of Definition (3). In this work, only the very near set of 3D cloud of points is deployed instead of processing the complete cloud of points (massive data sets). The vector field approach states a short territorial distance $r_{\alpha_{max}}$ to discriminate the objects beyond such a distance.

**Figure 13:**
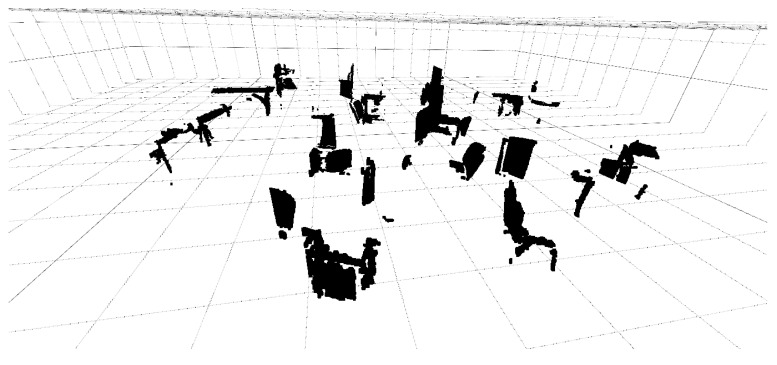
RGB-D data obstacles map (Robotics Lab) with discriminant criterion constraint (Definition (3)).

**Definition 3 (Obstacles Discriminant Condition).**  *The obstacles depth map is built by means of the next discriminant criterion*:

(56) $$p_{m}=\left\{p_{i}|\left(d_{s}<x<d_{l}\right)\cap \left(ds<y<d_{l}\right)\cap \left(d_{s}<z<d_{i}\right)\right\}$$

where $p_{m}={\left(x,y\right)}^{⊤}\in R^{2}$ are the local obstacle maps of the nearest points as illustrated in Figure 13. It is an experimental map of the robotics laboratory, with specific thresholds for $d_{l}$, $d_{s}$ and $d_{i}$.

From the current 3D map, the vertical Y-component is projected onto $R^{2}$ dimension, then the repulsive effects are yielded.

Therefore, the repulsive directional field function is defined in one-dimension by the next proposition,

**Proposition 7 (Repulsive Function).**  *The repulsive acceleration function exerted by α at distance $r_{\alpha }=∥p_{m}∥$ is defined by:*

(57) $$f_{\alpha }\left(x,y\right)=-\nabla_{x,y}\kappa_{\alpha }\left(k_{2}-r_{\alpha }\right)\cdot \cos\left(\phi_{\alpha }\left(\delta \right)\right)$$

*where the term $r_{\alpha }$ is the activation distance, $k_{2}$ is an obstacle territorial diameter, and $\kappa_{\alpha }$ is a constant gain of the acceleration amplitude in ms${}^{-2}$ that is set $\kappa_{\alpha }=1$ for analysis purpose. Likewise, the local obstacles distance is defined by:*

(58) $$r_{\alpha }=\sqrt[{2}]{x^{2}+y^{2}}=\sqrt[{2}]{{\left(x_{r}-x_{o}\right)}^{2}+{\left(y_{r}-y_{o}\right)}^{2}}$$

The angle $0\leq \phi_{\alpha }\leq \pi /2$ refers to the relation of sensed distance *δ*, and the distance $d_{l}\leq r_{\alpha_{max}}$ is established as the obstacle reaction distance.

**Definition 4 (Territorial Repulsive Distance).**  *The distance $d_{l}\leq r_{\alpha_{max}}$ establishes the obstacle repulsive acceleration reaction.*

(59) $$\phi_{\alpha }=\frac{\delta_{t}\pi }{2d_{l}}$$

by substituting the functional form for $r_{\alpha }$:

(60) $$f_{\alpha }\left(x,y\right)=-\nabla_{x,y}\left(k_{2}-\sqrt[{2}]{{\left(x_{r}-x_{o}\right)}^{2}+{\left(y_{r}-y_{o}\right)}^{2}}\right)\cdot \cos\left(\phi_{\alpha }\left(\delta \right)\right)$$

and algebraically expanding:

(61) $$f_{\alpha }\left(x,y\right)=\nabla_{x,y}\left[-k_{2}\cos\left(\phi_{\alpha }\left(\delta \right)\right)+\sqrt[{2}]{{\left(x_{r}-x_{o}\right)}^{2}+{\left(y_{r}-y_{o}\right)}^{2}}\cdot \cos\left(\phi_{\alpha }\left(\delta \right)\right)\right]$$

by applying the gradient operator $\nabla_{x,y}$, and simplifying algebraically:

(62) $$\frac{\partial f_{\alpha }}{\partial x}=\frac{\left(x_{r}-x_{o}\right)\cdot \cos\left(\phi_{\alpha }\left(\delta \right)\right)}{\sqrt[{2}]{{\left(x_{r}-x_{o}\right)}^{2}+{\left(y_{r}-y_{o}\right)}^{2}}}$$

and

(63) $$\frac{\partial f_{\alpha }}{\partial y}=\frac{\left(y_{r}-y_{o}\right)\cdot \cos\left(\phi_{\alpha }\left(\delta \right)\right)}{\sqrt[{2}]{{\left(x_{r}-x_{o}\right)}^{2}+{\left(y_{r}-y_{o}\right)}^{2}}}$$

finally, replacing $\sqrt[{2}]{{\left(x_{r}-x_{o}\right)}^{2}+{\left(y_{r}-y_{o}\right)}^{2}}$ by $r_{\alpha }$:

(64) $$\frac{\partial f_{\alpha }}{\partial x}=\frac{\left(x_{r}-x_{o}\right)\cdot \cos\left(\phi_{\alpha }\left(\delta \right)\right)}{r_{\alpha }}$$

and

(65) $$\frac{\partial f_{\alpha }}{\partial y}=\frac{\left(y_{r}-y_{o}\right)\cdot \cos\left(\phi_{\alpha }\left(\delta \right)\right)}{r_{\alpha }}$$

Thus, the repulsive vector field is provided by the following lemma,

**Lemma 4 (Repulsive Directional Vector Field).**  *The acceleration vector against α in terms of Cartesian components XY is:*

(66) $$f_{\alpha }\left(x,y\right)=\kappa_{\alpha }\frac{\cos(\phi_{\alpha })}{r_{\alpha }}\cdot \left(\begin{array}{c} x_{r}-x_{o}\\ y_{r}-y_{o} \end{array}\right)$$

**Figure 14:**
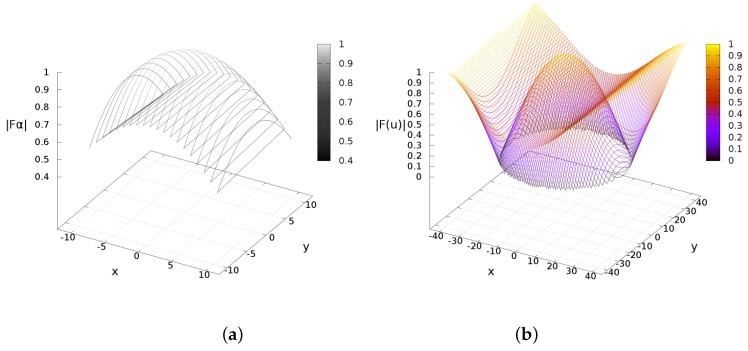
Repulsive acceleration behaviors. (**a**) Effects from $d_{l}\leq r_{\alpha_{max}}$; (**b**) Effects when $d_{l}>r_{\alpha_{max}}$.

Figure 14 depicts a simulation of the repulsive acceleration produced by the cosine field. Figure 14 depicts a simulation of the repulsive acceleration produced by the cosine field.

**Figure 15:**
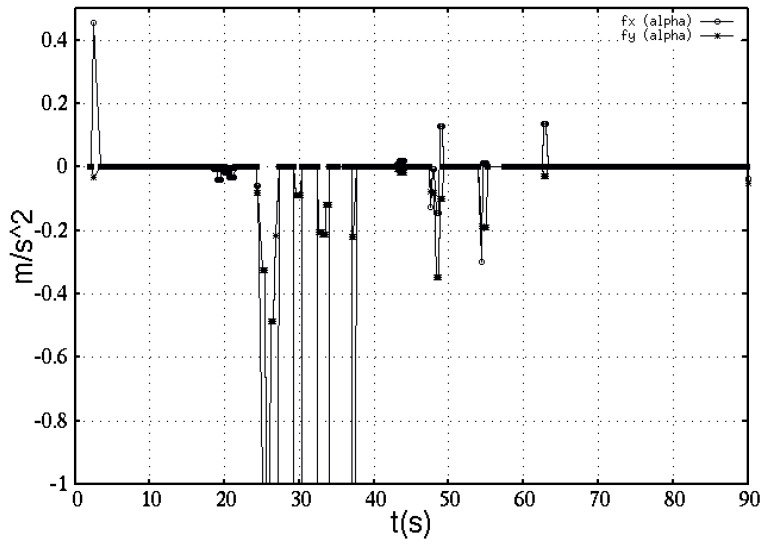
The robot’s acceleration components influenced by feed back from the RGB-D sensor, while detecting obstacles.

In addition, Figure 15 shows experimental behavior of the repulsive acceleration effects $f_{\alpha }$ w.r.t. the time t(s) taken by the robot to find the IR target. Obstacles detection are shown as impulse-like discontinuities occurring in components $f_{\alpha }^{x}$ and $f_{\alpha }^{y}$, which are detected within the robot’s territorial scope.

Due to the numeric scale shown, minor acceleration changes that are non visible occurred along the repulsive accelerations components.

## 8. Control Navigation Function

Previous Section 3 (IR nonlinear sensing model), Section 6 (motion planning model), and Section 7 (trigonometric directional derivatives) treated three main topics separately. Now, this section mathematically combines them in order to develop a control law that produces autonomous navigational missions for searching and exploring. According to the planning and directional derivative control schemes, robot’s posture is not relevant for our navigational framework. The vector field is organized by either first or second order derivatives, hence robot’s encoders are deployed to measure wheels’ rotational velocities. The encoder measurement model detects $n_{t}$ pulses with resolution of *R* pulses/rev for wheels of radius *r*, and it is stated by:

(67) $$\varphi =\frac{2\pi }{R}n_{t}$$

The direct wheels measurement provides angular instantaneous motion $\varphi_{t}$, and in terms of its first numeric derivative (central divided differences) [37], the following proposition is stated.

**Proposition 8 (Encoder Angular Velocity Observation).**  *A high-precision angular speed observation is obtained online by the first order differentiation:*

(68) $$\frac{d}{\mathrm{dt}}\varphi \left(t\right)=\frac{-\varphi (t+2)+8\varphi (t+1)-8\varphi (t-1)+\varphi (t-2)}{12\Delta t}$$

Therefore, the dual differential robot’s linear and angular velocities in terms of the wheels angular velocities are defined by:

(69) $$v_{t}=\frac{r}{2}\left(\frac{d}{\mathrm{dt}}\varphi_{r}+\frac{d}{\mathrm{dt}}\varphi_{l}\right)$$

and

(70) $$\omega_{t}=\frac{2r}{b}\left(\frac{d}{\mathrm{dt}}\varphi_{r}-\frac{d}{\mathrm{dt}}\varphi_{l}\right)$$

**Figure 16:**
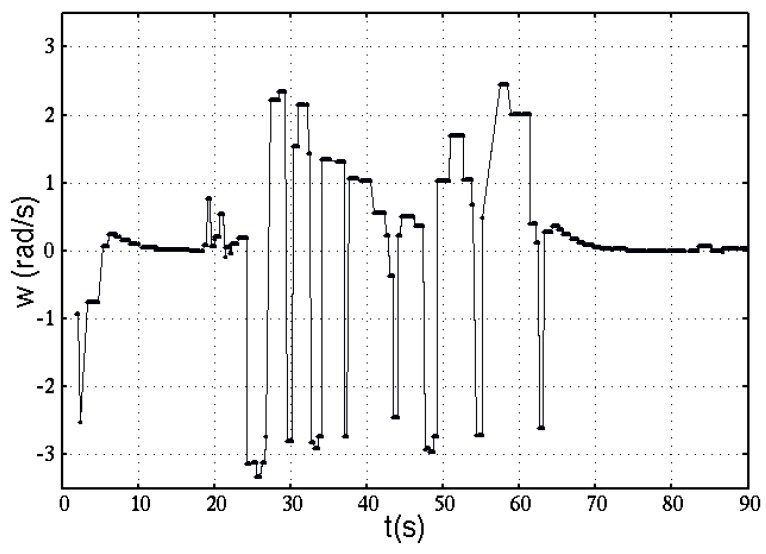
Robot’s angular velocity as inferred from encoders reading.

The real angular velocity during this experiment, is being depicted in Figure 16. Data were obtained during time t(s) of this experiment. The angular velocity is expressed in rad/s.

Thus, the robot’s instantaneous displacement $s_{t}$:

(71) $$s_{t}=s_{t-1}+\int_{t_{1}}^{t_{2}}v_{t}\mathrm{dt}$$

**Lemma 5 (Recursive Robot’s Position).**  *The instantaneous robot’s position is inferred recursively by:*

(72a) $$x_{r}=x_{t-1}+s_{t}\cos\left(\int_{t_{1}}^{t_{2}}\omega_{t}\mathrm{dt}\right)$$

*and*

(72b) $$y_{r}=y_{t-1}+s_{t}\sin\left(\int_{t_{1}}^{t_{2}}\omega_{t}\mathrm{dt}\right)$$

Therefore, the final equation for autonomous navigation control is established by:

**Theorem 2 (The Navigation Control Law).**  *The robot’s motion equation leading to γ while avoiding numerous α is defined by:*

(73) $$f_{T}=\kappa_{\gamma }\frac{-\sin(\phi_{\gamma })}{r_{\gamma }}\cdot \left(\begin{array}{c} x_{r}-x_{f}\\ y_{r}-y_{f} \end{array}\right)+\kappa_{\alpha }\sum_{\alpha }\frac{\cos(\phi_{\alpha })}{r_{\alpha }}\cdot \left(\begin{array}{c} x_{r}-x_{o}\\ y_{r}-y_{o} \end{array}\right)$$

The attractive and the repulsive magnitudes $∥f_{\gamma }∥$ and $\sum_{\alpha }∥f_{\alpha }∥$ interact together, and regardless of the repulsive acceleration behavior, the attractive acceleration simultaneously decreases successively. When obstacles were not detected, it produced a type of smooth nonlinear behavior from beginning to end. This behavior is due to the configuration parameters related to the obstacles’ distance set experimentally. The robot’s total velocity is obtained by a time integration of $f_{T}$, and a robot’s maximal real velocity constraint. The robot’s physical actuators reach until $v^{max}=1.35$ m/s with load included.

(74) $$\frac{dv_{t}}{dt}=f_{T}$$

completing differentials by integrations in both sides of the equation:

(75) $$\int_{v_{t}}^{v_{t+1}}dv_{t}=\int_{t_{1}}^{t_{2}}f_{T}dt$$

Thus, the new recursive form for velocity that is physically implementable in the robot is given next:

(76) $$v_{t+1}=v_{t}+f_{T}\left(t_{2}-t_{1}\right)$$

And the real permitted robot’s speed ${\hat{v}}_{T}$ is constrained by the next expression:

(77) $${\hat{v}}_{T}=\left\{\begin{array}{cc} v_{t+1}, & ∥v_{t+1}∥\leq v^{max}\\ \frac{v_{t+1}}{v^{max}}, & \mathrm{otherwise} \end{array}\right.$$

**Figure 17:**
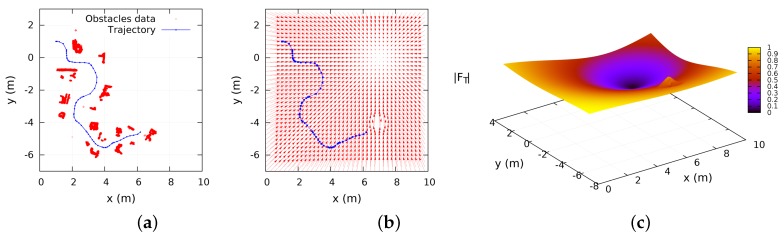
Experimental attractive/repulsive effects and obstacles map. (**a**) 2D map using 1.2 m of territorial scope; (**b**) vector field $f_{\gamma }$ and $f_{\alpha }$ in current robot’s observation; (**c**) 3D surface of $f_{\gamma }$ and $f_{\alpha }$ in current robot’s observation.

Figure 17a depicts experimental results of the robot’s trajectory in the obstacles map according to the Equation (2). Figure 17b depicts the repulsive and attractive vector field. Figure 17c illustrates the same vector field as 3D surface. The attractive effects produced by the source of heat at position (7,0) are demonstrated in the same illustration.

## 9. Experimental Navigation

**Figure 18:**
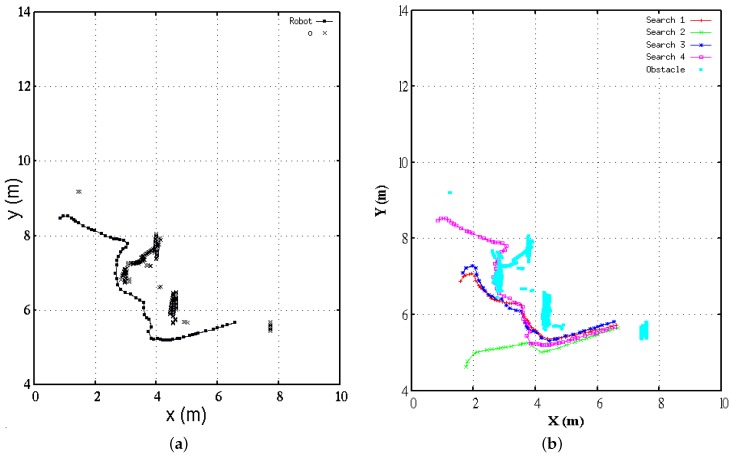
(**a**) Robot’s experimental search task, a trajectory is yielded while finding an IR radiation source; (**b**) Multiple experimental search missions starting at different locations.

This section presents some discussions on further experimental results and parameters numerical adjustments. The experiments’ purpose is to autonomously find a near-IR source within the Robotic Lab in the presence of multiple obstacles. This manuscript’s mathematical framework provided us concrete and sophisticated elements to develop a control-sensing algorithm. The Appendix A lists the robot’s pseudo code that the robotic platform carried out for experimental autonomous missions. A successful experimental search is depicted by the robot’s trajectory in Figure 18a. The obstacles mapped are denoted by the symbol ×, and only appear on segments of the obstacles because RGB-D data were constrained using a spatial discriminant filter. In this work’s purpose, obstacles detection is useful solely to avoid online collisions with too near obstacles, unlike other approaches, where a more extensive local map is constructed and used for planning or environmental modeling.

The IR sensing model is calibrated a priori before sensor deployment. Calibration and characterization are required for any new source of IR emissions (i.e., different temperature, device’s material), and for any change to the sensing device (e.g., changing the filter film, using different camera or lens). For the case of experiment of Figure 18, our IR sensor is a USB camera with vertical visual angle $37.8^{\circ }$, and horizontal angle $54.4^{\circ }$. The exponential model values were set to α=171.768, and β=−0.0128616, and, likewise, for the polynomial coefficients $a_{0}=192.261$, $a_{1}=-2.95007$, and $a_{2}=0.0158797$. In addition, the control parameters were established as: an attractive territorial distance $r_{\gamma }max=15$ m, a repulsive territorial distance $r_{\alpha }max=1$ m. Discriminant constraints $d_{s}=1$ m and $d_{i}=0$, which means that only objects on the line of sight of 1 m were detected. The robot’s encoders have a resolution R=500 pul/rev. An attractive acceleration gain $\kappa_{\gamma }=8$ m/s${}^{2}$, and repulsive gain $\kappa_{\alpha }=1\;$m/s${}^{2}$. The physically allowed maximal speed of the robot is $v^{max}=1.3$ m/s. It is worth mentioning that any weight change onboard the robot implies parameter re-adjustments.

**Figure 19:**
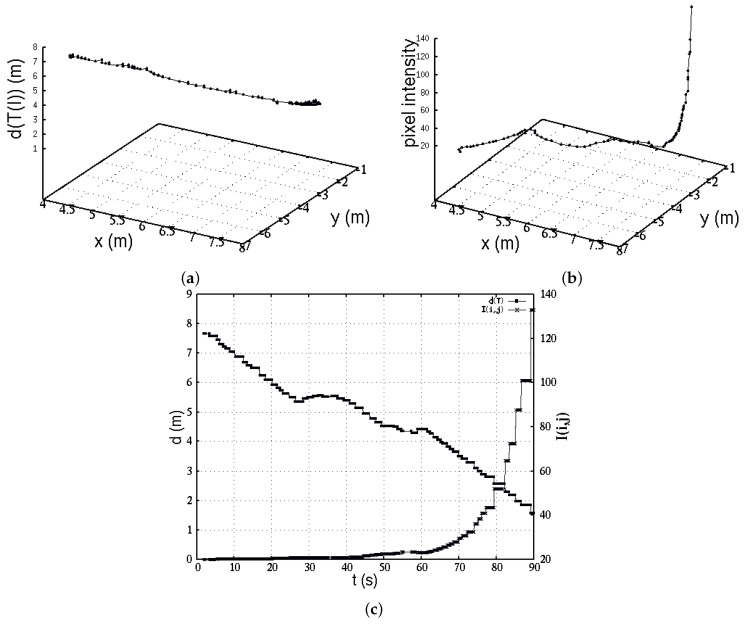
(**a**) Distance $∥(x_{f},y_{f})∥$ towards the IR radiation source; (**b**) Robot’s observations, pixel intensity of interest $I_{M}\left(i,j\right)$ at each robot’s location; (**c**) Comparative data, $I_{M}$ and ${\left(x_{f}^{2}+y_{f}^{2}\right)}^{1/2}$.

The solution of the planning system is validated by results depicted in Figure 19. The successive numerical approximations about distance estimation are plotted at each robot’s Cartesian position. During navigation, the IR sensor’s field of view was able to see either direct device near-IR radiance, or other objects’ reflectance. These situations happened due to the robot’s turns. However, it really did not represent a major problem for the robot’s visual sensor because of the high radiance power of the IR source. At each control loop, the vision algorithm calculated maximal 5% of the pixels’ intensity. Nevertheless, only a single region was considered for pixel processing. For future works, however, the algorithm must take into account multiple regions simultaneously that are detected with similar levels of radiance. The vision algorithm could even better enhance the robot’s efficacy to find the IR source, however, currently, this is out of the scope of this work.

Therefore, Figure 19b shows the maximal intensity pixel value w.r.t. the robot’s Cartesian location. As the robot gets nearer to the IR radiation source, the pixel intensity observation value increases. Sometimes, this occurs when the robot is unable to see directly towards the IR source; in that case, the robot utilizes prior numeric values of pixel observation. If numerical inaccuracy is evolving, then it is successively corrected when the robot again detects the IR source.

In addition, Figure 19c depicts a comparative view of behaviors between sensor data $I_{M}$, and estimation of $\left(x_{f},y_{f}\right)$. It is interesting to notice the period 60 s–90 s, where the distance is linearly decaying, and the intensity $I_{M}$ increases rapidly. This means the robot is sensing the IR source directly in its field of view, and therefore leading straight towards the destination. As an evidence of this approach, there is an important correlation with the robot’s trajectory of Figure 18, where along the *X*-axis in range 4 m–7 m the robot basically moved in a straight line. Finally, we validated our approach through multiple experiments with different robot’s initial positions, and minimal parameters adjustment. Figure 18b illustrates four different search and find trajectories carried out by the wheeled robot. Trajectory 4 is the pathway that was previously used for analysis through this manuscript. In this plot, experiments began with the robot at different Cartesian locations, but kept the IR source at the same location for the four experiments.

## 10. Conclusions

An autonomous navigation system for a mobile robot to find a thermal source by sensing IR images was developed. This manuscript has focused on the mathematical modeling and deterministic formulations of the robotic system. The system performance was fast, precise, and adequate for online and real-time applications. A passive thermal infra-red visual sensor was developed in the laboratory in a way that was very low-cost and effective for the task’s purpose. The sensor’s capability to detect the IR spectrum was characterized. By using Wein’s law of displacement of radiation power, the infra-red images were coherent with the family of curves established and was solved by online adaptive threshold for image segmentation. Empirical models were fitted by nonlinear regressions, 2nd degree polynomial for image-temperature, and exponential for image-distance. Both models adjusted precisely and conveniently including environmental degraded atmospheric transmission, varying lighting and thermal conditions, lens’ diffraction pattern, and so forth. Thus, by following the proposed methodology, both models can be automatically recalibrated either when changing scenarios, or changing the CCD camera device. The subtle closed-form of the sensing model distance-temperature, and inverse solution fitted a family of curves. If parameters re-adjusted, it allowed the distinguishing of different thermal sources with the same IR image.

The proposed searching planning system $d{\left(T\right)}_{t}^{2}={\left(x_{f}-x_{t}\right)}^{2}+{\left(y_{f}-y_{t}\right)}^{2}$ was solved using an online numerical solution, which computationally faster to implement than the analytical solution. The present approach expended less than 10 iterations at each control loop to estimate the heat source location in global coordinates.

For the robot’s course control, we formulated a simple but effective half sine (attractive) and half cosine (repulsive) functions as directional derivatives. The control activation distance is a range between $\left(0,\cdots ,\frac{\pi }{2}\right)$rad. Such an angle is determined as a function of the robot’s territorial distances w.r.t. the goal and the obstacles.

Finally, the proposed approach can be applied to search other types of targets without making considerable changes and developments. For instance, the planner and the navigation control may prevail as proposed in this manuscript. However, in order to search and explore in other types of real environments and applications, the major changes focus on obtaining new empirical sensor models, and reformulating the sensing models for different types of sensors. Other sensors may vary in types and modalities such as electromagnetic radiation sources, electric potentials, electrochemical signals. In the meantime, a proper sensing device is available and is modeled by applying the same regression methods.

## Figures and Tables

**Table 1:** IR sensor characterization detecting radiation.

T(${}^{\circ }$C)	T(${}^{\circ }$K)	*λ*(nm)	$I_{M}\left(i,j\right)$
248.76	521.91	5552.68	39
260.78	533.93	5427.68	52
270.92	544.07	5326.52	71
283.54	556.69	5205.77	100
289.56	562.71	5150.08	118
301.48	574.63	5043.25	157

## Supplementary Materials

The following videos are available online at https://www.youtube.com/watch?v=Cs4tt0AoIFo with title ”Robot Control and Planning Navigation for Heat-Source Finding”; and, https://www.youtube.com/watch?v=MKMzvyDCjtw with title ”Measuring 3D Position of a Thermal-source by a Home-made IR-Camera”.

## Appendix A. Control and Search Algorithm

The general algorithm that was implemented for the robot platform is simplified and listed below. The pseudo code contains the three main parts: use of the thermal sensing model, the recursive planner algorithm, and directional field for navigation control.

**Algorithm 1:** IR emission search and exploring general robot’s algorithm.
